# Controllable Pulse Parameter TMS and TMS-EEG As Novel Approaches to Improve Neural Targeting with rTMS in Human Cerebral Cortex

**DOI:** 10.3389/fncir.2016.00097

**Published:** 2016-11-29

**Authors:** Ricci Hannah, Lorenzo Rocchi, Sara Tremblay, John C. Rothwell

**Affiliations:** Sobell Department of Motor Neuroscience and Movement Disorders, UCL Institute of NeurologyLondon, UK

**Keywords:** non-invasive brain stimulation, controllable pulse parameter TMS, TMS pulse duration, TMS current direction, TMS-EEG

## Introduction

Repetitive transcranial magnetic stimulation (rTMS) can produce after-effects on the excitability and function of the stimulated site cortical site that outlasts the period of stimulation for several minutes or hours (Huang et al., 2005; Hamada et al., 2008; Ridding and Ziemann, 2010; Sommer et al., 2013). These are thought to involve early phases of long term potentiation/depression at cortical synapses. Depending on the area stimulated, the after-effects can influence performance of a variety of cognitive and motor tasks, as well as learning (Censor and Cohen, 2011; Parkin et al., 2015). Reports of beneficial effects on behavior in healthy populations have led to widespread interest in applying rTMS therapeutically, for example in patients with neuropsychiatric and neurological disorders (Ridding and Rothwell, 2007; George et al., 2013; Lefaucheur et al., 2014).

A major issue with rTMS protocols is that the effects vary considerably within and between individuals (Maeda et al., 2000; Hamada et al., 2013; Vernet et al., 2013; Goldsworthy et al., 2014; Hinder et al., 2014; López-Alonso et al., 2014; Vallence et al., 2015; Simeoni et al., 2016), which causes problems in replication of results in a research setting (Héroux et al., 2015), and is an obstacle to using rTMS in a therapeutic setting. A separate, but related, issue is that rTMS over a given cortical area is often assumed to affect all neuronal populations equally and thus affect all behaviors involving that area similarly, but this may not be true. Here we argue that advanced technologies and methodologies, such as controllable pulse parameter TMS (cTMS; Peterchev et al., 2014) and combining TMS with electroencephalography (EEG; Ilmoniemi and Kicic, 2010), might facilitate the development of more selective forms of stimulation targeting particular neuronal populations or brain states, and ultimately improve the reliability and behavioral specificity of rTMS protocols.

## Variability of rTMS after-effects

After rTMS applied to the motor cortex (M1), intra- and inter-individual variability of plasticity-like after-effects are usually measured in terms of the change in motor evoked potential (MEP) amplitude (Huang et al., 2005; Hamada et al., 2008; Ridding and Ziemann, 2010; Sommer et al., 2013). Recent studies suggest that, at least for some rTMS protocols (i.e., intermittent and continuous theta burst stimulation), the variance in outcomes accounted for by intra-individual variability is less than a third of that due to inter-individual variability (Hinder et al., 2014; Vallence et al., 2015). The inter-individual variability is, however, quite substantial and evidence suggests most protocols only produce the “expected” effects in 40–60% participants (Hamada et al., 2013; Hinder et al., 2014; López-Alonso et al., 2014; Vallence et al., 2015; Simeoni et al., 2016). Exploration of factors contributing to inter-individual differences has shown that they involve genetics, sex, age, physical fitness, hormone levels, and brain anatomy (Ridding and Ziemann, 2010). We recently suggested that individual differences in response to rTMS may also arise because different populations of cortical neurons are more readily recruited or are more excitable in different people and at different times (Hamada et al., 2013). Thus one potential solution to the problem of variability is to improve the selectivity of TMS pulses to ensure optimal targeting of relevant neuronal populations.

## TMS pulse parameters influence neural targeting and response to rTMS in M1

The MEP evoked by stimulation over M1 is thought to be generated by activation of the axons of excitatory synaptic inputs to corticospinal neurons (CSNs). It is well-known that TMS with posterior-anterior (PA) induced currents in the brain, i.e., oriented perpendicular to the central sulcus, evokes corticospinal activity and MEPs with a lower threshold and shorter latency than with anterior-posterior (AP) currents, i.e., oriented at 180° with respect to PA pulses (Day et al., 1989; Di Lazzaro et al., 2001; Hamada et al., 2013). The implication is that the two directions activate different sets of neurons in M1, PA-sensitive and AP-sensitive inputs. This is important because these inputs are known to have different physiological properties, responding differently to short-interval intracortical inhibition between two closely spaced TMS pulses (Hanajima et al., 1998) and to somatosensory input (Tokimura et al., 2000; Hannah and Rothwell, in press). Moreover, modeling (Salvador et al., 2011) and connectivity (Volz et al., 2015) studies suggest these inputs may arise from different cortical regions (including premotor and somatosensory), implying that they may be engaged in different aspects of motor behavior. However, whilst PA pulses typically recruit early inputs, the ability of AP pulses to selectively recruit late inputs still varies between people, being very clear in some and lacking in others (Di Lazzaro et al., 2001; Hamada et al., 2013).

The recruitment threshold of a stimulated axon depends not only on the direction of the stimulating current, but also on the current intensity and duration of stimuli. Axons possess different strength-duration properties, and in peripheral nerves this means that particular sets of axons (sensory or motor) can be targeted by varying the pulse width of electrical stimuli (Mogyoros et al., 1996). Commercially-available TMS devices cannot vary pulse width and cannot make use of the different strength-duration behavior of cortical axons to target particular sets of neurons. However, novel devices such as the cTMS (Peterchev et al., 2014) allow flexible control of pulse width, shape (e.g., monophasic/unidirectional or biphasic/bidirectional) and direction, and can be implemented during single pulse and rTMS modes. We recently showed that motor cortical axons recruited by AP and PA currents have different strength-duration behavior (D'Ostilio et al., 2016). We also found that AP TMS with a short duration pulse (30 μs; AP_S_) much more reliably activates different sets of inputs than PA TMS with a long duration pulse (120 μs; PA_L_). This allows stimulation to be focused more clearly on different sets of inputs and, importantly, the approach can be applied during high frequency rTMS protocols.

Many rTMS protocols apply stimuli at rates of 1 Hz or more (Huang et al., 2005; Hamada et al., 2008; Sommer et al., 2013). Conventional rTMS machines produce high frequency stimulation by generating a biphasic current pulse rather than the monophasic stimulus used in single pulse experiments. We previously argued that this would activate at least two different cortical circuits (PA- and AP-sensitive inputs) and produce a mixture of effects that would contribute to inter-individual variation in outcome (Hamada et al., 2013). Thus, we expect that applying rTMS protocols with more selective unidirectional pulses ought to produce clearer and more reproducible effects on MEPs. For example, a recent study showed that inhibition after 1 Hz rTMS delivered via a cTMS device is best achieved with a monophasic stimulus waveform compared with standard sinusoidal biphasic pulse (Goetz et al., 2016). Preliminary evidence suggests that higher frequency types of rTMS such as intermittent and continuous TBS (iTBS and cTBS) protocols are also sensitive to pulse parameters. Traditional iTBS and cTBS applied with biphasic pulses tend to facilitate or inhibit M1 excitability, respectively (Huang et al., 2005). However, when applied with unidirectional AP_S_ and PA_L_ pulses, there is preliminary evidence to suggest that the effects on M1 excitability are determined more by the pulse type, and thus potentially the specific neural population recruited, rather than the pattern of TBS (intermittent or continuous; Hannah et al., 2014; Sommer et al., 2014). Larger investigations are currently underway to compare the efficacy of standard TBS protocols with new targeted TBS protocols in terms of the magnitude and inter-individual variability of changes in both neurophysiological (i.e., MEPs) and behavioral (i.e., motor performance and learning; *see below*) outcomes.

## Directionally selective neurons participate in different behaviors

A caveat in relation to MEP measurements is that changes induced by rTMS may not be the most functionally relevant outcome. Research and therapeutic applications often require reproducible effects on behavior and these may be unrelated to effects on MEPs. For instance two conceptually alike rTMS protocols may produce similar effects on MEPs, yet each could elicit distinct effects on different forms of motor behavior. Therefore, there is a need to develop protocols that not only produce less variable effects within and between individuals, but also have clearly defined effects on behavior.

For example, we recently employed an indirect method of targeting and pre-conditioning AP- and PA-sensitive neurons via two paired associative stimulation (PAS) protocols, to investigate whether they participated in different motor behaviors (Hamada et al., 2014). A protocol that putatively increased excitability of PA-inputs interacted with learning in a ballistic thumb acceleration task (model-free learning), whereas one that increased excitability of AP-inputs interacted with learning in a visuomotor adaptation task (model-based learning). One prediction from this is that directly conditioning the excitability of AP- or PA-inputs with unidirectional rTMS, rather than the previous indirect PAS methods, would produce selective effects on motor behavior. The implication would be that that this method may produce more selective effects on motor behavior than has previously been possible. Experiments are currently underway in our laboratory to test this possibility.

## Achieving more selective stimulation in non-motor cortical areas

rTMS is frequently applied to non-motor areas of the cortex, to study their function and to intervene therapeutically, as in depression where stimulation is applied to the dorsolateral prefrontal cortex (Ridding and Rothwell, 2007; Lefaucheur et al., 2014). Given the similarities in organization of all regions of neocortex (Mountcastle, 1997), we expect that similar principles concerning the anisotropy and waveform dependence of stimulation also apply to areas of cortex besides M1. Thus it should be possible to achieve more selective stimulation of other cortical regions. For example, preliminary evidence suggests that premotor and prefrontal regions are sensitive to direction of the TMS induced-current. Premotor-primary motor connectivity, assessed via a twin-coil paradigm, was most evident when using an AP-directed conditioning stimulus, as opposed to PA (Civardi et al., 2001). In prefrontal cortex, PA directed rTMS was shown to induce larger changes in blood oxygenation detected with near-infrared spectroscopy vs. AP and latero-medial coil orientations (Thomson et al., 2013). However, further studies with more direct measurements of cortical activity are needed to confirm that model of M1 responsiveness to TMS is valid across the whole cerebral cortex.

To this purpose, combining TMS and EEG might be useful since it allows direct measurement of cortical responses to TMS pulses (Ilmoniemi and Kicic, 2010), providing detailed descriptions of TMS evoked potentials (TEPs) in terms of the average evoked activity and the effects of TMS on ongoing EEG rhythms. These different aspects of the cortical response to TMS are thought to reflect specific electrophysiologic and pharmacological characteristics. Different components of TEPs may reflect activity in distinct subset of cortical neurons. In this regard, Bonato et al. (2006) showed that when TMS was delivered over M1 at a coil orientation that is sub-optimal for eliciting MEPs (135°), the lack of MEPs was paralleled by the absence of some early components of TEPs (N15, P30) (Bonato et al., 2006). Together with data showing that these components also correlate with MEP amplitude (Mäki and Ilmoniemi, 2010), these studies suggest some early TEP components may be caused by TMS-induced activation of directionally-sensitive motor cortical neurons. The implication is that particular TMS-EEG responses may be affected by TMS pulse parameters such as direction, and thus could help confirm that the rules governing sensitivity to stimulation in M1 apply to other cortical areas. This might lend to the development of more selective forms of rTMS in non-motor areas of the cortex.

## Closed-loop stimulation with TMS-EEG

In addition to probing the selectivity of TMS for different neural populations, EEG might be useful to precisely time TMS pulses according to different states of cortical excitability. It is known that excitability of M1 (Sauseng et al., 2009; Mäki and Ilmoniemi, 2010; Keil et al., 2014) and the primary visual cortex (Romei et al., 2008) are dependent on the phase of ongoing oscillatory activity or to cortical dynamics with a broader temporal span, such as event-related desynchronization (ERD) in the beta band (Schulz et al., 2014). This means that randomly delivered TMS pulses likely stimulate populations of neurons in different functional states. Consequently, attempts have been made to tune TMS pulses to different brain events detectable with the EEG, namely closed-loop stimulation. Stimulation can be synchronized relative to endogenous or externally triggered EEG activity, such as power in specific spectral bands, latency or amplitude of evoked responses, and phase of oscillatory activity (Bergmann et al., 2016). Kraus and coworkers suggested that it is possible to modify cortical excitability by delivering TMS pulses during ERD in the beta band during a motor imagery task (Kraus et al., 2016). This effect was not observed when the pulses were delivered at rest, although it remains to be determined if TMS delivered during imagery, but outside periods of beta ERD, would produce a similar effect. The closed-loop approach could also be used to tune common rTMS paradigms to endogenous or behaviorally-relevant cortical oscillations, allowing the development of individualized protocols. In a recent study, Brownjohn et al. (2014) demonstrated that tuning iTBS according to the dominant frequency of the stimulated cortex was as effective as standard iTBS in increasing MEPs amplitude. Although a clear advantage of this individualization was not demonstrated, this approach might be further developed to enhance the specificity of rTMS protocols for physiologically- and behaviorally-relevant neural populations, enabling us to probe the function of these circuits in more detail.

TMS-EEG may also provide a more comprehensive readout of rTMS effects on cortical excitability, since different indices, such as TEPs and changes in the oscillatory activity may reflect different mechanisms (Chung et al., 2015). Several aspects of the EEG signal have been shown to be markers of the effect of rTMS or TBS, such as an increase in delta wave power (Assenza et al., 2015), changes in power in the theta and alpha bands (Vernet et al., 2013) and changes in the amplitude or latency of specific components of TEPs (Van Der Werf and Paus, 2006; Vernet et al., 2013; Casula et al., 2014). In addition, EEG and MEPs may be differentially sensitive to changes in neural activity induced by rTMS. For example, one study reported a different time course of effects of cTBS on EEG vs. MEPs (Noh et al., 2012). Thus EEG and TMS-EEG may provide distinct information from MEPs and offer insights into the physiological response of cortical areas to rTMS.

## Delimiting the parameter space for rTMS

The development of novel technologies and techniques such as cTMS and TMS-EEG dramatically increases the parameter space for rTMS protocols (e.g., pulse shape, direction, intensity, frequency, brain state, etc.). Adopting a “look-and-see” approach is likely to be inefficient approach to narrowing the parameter space. Furthermore, by choosing stimulation parameters at random, no information would be gained on the mechanism of action. This would offer little insight into how to improve a protocol or predict for which applications it is optimal. At present, data are probably insufficient to provide a step-by-step guide to developing rTMS protocols based on the newly mentioned technical possibilities. However, we have already discovered in our recent work with single cTMS pulses that two very different sets of stimulus parameters (AP_S_ and PA_L_) activate quite separate populations of cortical neurons (D'Ostilio et al., 2016), and we have selected these as the starting point for our rTMS studies probing the neurophysiological (Hannah et al., 2014; Sommer et al., 2014) and behavioral outcomes. Hopefully, knowledge gained from this and other studies (e.g., TMS-EEG) will allow us to further develop structured and hypothesis-driven approaches.

## Conclusions

Research in the human M1 suggests that employing advanced TMS devices to manipulate shape and directionality of the stimulus waveform allows more control over the neural populations activated by a TMS pulse. This may allow us to study in detail their roles in behavior. Techniques such as TMS-EEG may be useful in confirming that similar control of TMS pulse parameters produces more selective stimulation in other cortical areas, and open those areas up to detailed study with more targeted forms of rTMS.

## Author contributions

Substantial contributions to the conception or design of the work (RH, LR, ST, JR); drafting the work or revising it critically for important intellectual content (RH, LR, ST, JR); final approval of the version to be published (RH, LR, ST, JR); agreement to be accountable for all aspects of the work in ensuring that questions related to the accuracy or integrity of any part of the work are appropriately investigated and resolved (RH, LR, ST, JR).

## Funding

RH and JR were supported by a Medical Research Council grant (MR/K01384X/1). ST was supported by a Canadian Institutes of Health Research fellowship award.

### Conflict of interest statement

The authors declare that the research was conducted in the absence of any commercial or financial relationships that could be construed as a potential conflict of interest. The reviewer MS and handling Editor declared their shared affiliation, and the handling Editor states that the process nevertheless met the standards of a fair and objective review.
